# Ilaprazole and Clopidogrel Resistance in Acute Stroke Patients

**DOI:** 10.3390/biomedicines10061366

**Published:** 2022-06-09

**Authors:** In Hwan Lim, Seung Jae Lee, Byoung-Soo Shin, Hyun Goo Kang

**Affiliations:** 1 Department of Pharmacology, School of Medicine, Wonkwang University, Iksan 54538, Korea; inhwanlim@naver.com; 2 Department of Chemistry, Institute for Molecular Biology and Genetics, Jeonbuk National University, Jeonju 54907, Korea; slee026@gmail.com; 3 Department of Neurology, Jeonbuk National University, Jeonju 54907, Korea; sbsoo@jbnu.ac.kr; 4 Research Institute of Clinical Medicine, Jeonbuk National University, Jeonju 54907, Korea; 5 Biomedical Research Institute, Jeonbuk National University Medical School and Hospital, Jeonju 54907, Korea

**Keywords:** clopidogrel, clopidogrel resistance, drug-drug interaction, ilaprazole, ischemic stroke, proton pump inhibitor, VerifyNow

## Abstract

Clopidogrel, an antiplatelet agent used for secondary prevention of cerebrovascular diseases, is often taken with proton pump inhibitors (PPIs). Generally, the combined use of clopidogrel and PPIs causes adverse drug–drug interactions. VerifyNow is a quick and convenient method to confirm clopidogrel resistance (CR), which compromises adequate antithrombotic effects. We aimed to confirm CR, identify its factors, and determine the influence of the combination of ilaprazole and clopidogrel on clopidogrel using VerifyNow. In this retrospective study, we examined patients who were receiving clopidogrel after three months, starting within one week from the onset of cerebral infarction symptoms. Clinical records, imaging records, and diagnostic laboratory results, including P2Y12 reaction units (PRU), were compared and analyzed to check for CR. Additionally, the groups treated with either both ilaprazole and clopidogrel or with medications other than ilaprazole were comparatively analyzed. CR was defined as a PRU ≥240 after clopidogrel for three months. Among factors influencing CR by affecting clopidogrel metabolism, positive statistical correlations with age and alcohol consumption were confirmed. The diagnostic tests revealed a lower glomerular filtration rate and platelet count of the CR-positive group. This finding proved that the combination therapy of ilaprazole and clopidogrel is safe, as it does not interfere with the metabolism of clopidogrel.

## 1. Introduction

Cerebral infarction, myocardial infarction, and transient ischemic attack are commonly known as ischemic cerebrovascular diseases. Although their mortality rates are decreasing, they can debilitate the quality of life after onset and have a high risk of recurrence. Ischemic stroke is still the cause of many deaths, disabilities, and resulting social burden worldwide [1]. Imaging study, such as brain computed tomography (CT), CT angiography, or magnetic resonance image, is required to investigate sudden neurological symptoms, such as unilateral hemiparesis, aphasia, dysarthria, and paresthesia, which are typical symptoms of stroke. For revascularization in acute stroke, treatment of intravenous thrombolysis and intra-arterial thrombectomy is required [2]. After that, anticoagulation or antithrombotic agents are used for primary and secondary prevention of these diseases, depending on the cause [3,4]. Ischemic stroke is divided into causes, such as large artery atherosclerosis, cardioembolism, and small vessel disease. Various antiplatelet agents are commonly used for noncardioembolic ischemic stroke [5]. Clopidogrel, belonging to the thienopyridine family, is one of the most used agents in the clinical setting. Clopidogrel is a critical component and is used not only for mono antiplatelet therapy but also for dual antiplatelet therapy (DAPT) [6]. Among patients using clopidogrel, those receiving DAPT, those with gastrointestinal (GI) bleeding, or those using steroids or nonsteroidal anti-inflammatory drugs are additionally required to take proton pump inhibitors (PPIs) [7].

Clopidogrel and PPIs utilize cytochrome P450 (CYP450) in the liver during the metabolic process [8,9]. Using these drugs simultaneously may lower the antithrombotic effect as they interfere with each other’s metabolism. Ilaprazole is a PPI that has begun to be used relatively recently. It is known to have little interaction with CYP450 [10]. Only a small portion of ilaprazole is metabolized to CYP3A4 and CYP2C19, which belong to the CYP450 family [10]. Although the exact mechanism has not yet been determined, it was confirmed that ilaprazole is metabolized through a nonenzymatic reaction [10]. Consequently, it is presumed that the combined treatment of ilaprazole and clopidogrel does not affect the activity and metabolism of clopidogrel. In vitro experiments showed that clopidogrel and ilaprazole do not affect metabolism [11], but studies have not been conducted on the combined use of ilaprazole and clopidogrel in acute cerebral infarction and the resulting clopidogrel resistance.

Clopidogrel exhibits antiplatelet effects by inhibiting ADP-induced platelet activation upon selectively blocking the P2Y_12_ receptor [12]. Clopidogrel resistance (CR) occurs when clopidogrel is administered to a patient and does not properly act as an antiplatelet [13]. CR can be confirmed by the presence of P2Y_12_ activity and ADP-induced platelet aggregation, even following sufficient administration of clopidogrel [13]. Assay methods that have been used to evaluate the effectiveness of clopidogrel are light-transmission aggregometry, multiplate analysis, platelet function analyzer-100, VerifyNow, thromboelastography platelet mapping, and flow cytometry vasodilator-stimulated phosphoprotein phosphorylation [14]. The VerifyNow method has several advantages: it is simple, fast, requires a small sample volume, and checks point-of-care status [15].

This study’s primary objective was to identify the factors that affect CR by influencing the pharmacological action of clopidogrel. The secondary objective was to use VerifyNow to evaluate whether the activity of clopidogrel reduced when concurrently administering clopidogrel and ilaprazole, which could easily confirm CR. The results of this study are expected to help identify a combination treatment method that has fewer side effects and effectively facilitates the action of each drug.

## 2. Materials and Methods

### 2.1. Patients

In this retrospective study, we examined patients aged 20 years or older who were diagnosed with cerebral infarction at the hospital within seven days of symptom onset from 1 April 2019 to 31 May 2021. Patients who received clopidogrel as a treatment for acute cerebral infarction during hospitalization and had been continuously receiving clopidogrel for three months, even after the onset of cerebral infarction, were enrolled. The study patients had a P2Y_12_ reaction unit (PRU), and the inhibition percentage results at the time of admission and three months after the onset of cerebral infarction were determined using VerifyNow (Accriva Diagnostics, San Deigo, CA, USA). This study excluded patients who had been experiencing cerebral infarction symptoms for more than seven days at the time of hospital arrival, who did not have VerifyNow results at the time of admission, in whom outpatient follow-up was difficult to conduct, or who did not have VerifyNow results after 3 months. This study also excluded patients who took antiplatelet agents other than clopidogrel, changed their medications from clopidogrel to another antiplatelet agent during follow-up, and did not receive full stroke evaluation, such as brain imaging and bloodwork.

### 2.2. Data Collection and Definition

Although data in the forms of clinical and imaging records using a registry database and medical records were retrospectively examined, CR was defined as a PRU ≥240 after three months of clopidogrel use [16]. Hypertension, diabetes mellitus (DM), hyperlipidemia, coronary artery disease (CAD), alcohol consumption, and smoking were examined in each group as factors that can affect CR. Hypertension patients were defined as those who had been previously diagnosed with hypertension and were taking medications or those whose blood pressure was ≥140/90 mmHg during hospitalization. DM patients were defined as those who had previously been diagnosed with DM and were taking medication, had postprandial blood glucose levels of ≥200 mg/dL two hours after a meal, had fasting blood glucose levels of ≥126 mg/dL, or had hemoglobin A1C levels of ≥6.5. Hyperlipidemia was defined as a total cholesterol level of ≥240 mg/dL, triglyceride level of ≥200 mg/dL, low-density lipoprotein of ≥160 mg/dL, or high-density lipoprotein of 40 mg/dL or lower. CAD was defined in patients who had been diagnosed with CAD and were taking medication, had a history of the interventional procedure, or had a history of bypass surgery, such as coronary artery bypass graft. The alcohol intake of patients was assessed using a variety of questions. Patients who drank more than 12 times per year (10 g or more of alcohol per time) were defined as alcohol drinkers. If participants responded that they did not drink or they drank less than 12 times per year (10 g or more of alcohol per time), they were defined as nonalcohol drinkers in this study [17,18]. Smoking, for purposes of this study, was defined as smoking at the time of admission or quitting smoking within one year from the date of admission. The statuses of patients at the time of admission were assessed using the National Institutes of Health Stroke Scale and the modified Rankin Scale (mRS) to compare neurological severity. Additionally, recurrent stroke rates and diagnostic test results were compared.

### 2.3. Statistical Analysis

First, we divided patients into a CR-positive group and a CR-negative group and compared age, sex, risk factors, and the clinical outcomes between groups. Nominal variables were comparatively analyzed using chi-square and Fisher’s tests, and continuous variables were comparatively analyzed using the t-test and Wilcoxon rank-sum analysis. We confirmed mutually influencing factors by conducting a multivariate regression analysis using the factors (*p* ≤ 0.1) identified from these analyses. Second, we divided the patients taking clopidogrel into those who took ilaprazole and those who took GI-protective medications other than ilaprazole and then compared their age, sex, risk factors, and clinical results. These two groups were separately compared for nominal and continuous variables, as was conducted in the previous analysis. Statistical significance was determined with a *p*-value of ≤0.05. All analyses were performed using SPSS (version 21.0; IBM Corporation, Armonk, NY, USA).

## 3. Results

### 3.1. Factors Affecting CR

CR was defined as a PRU ≥240 after clopidogrel administration for 3 months after the onset of acute cerebral infarction [19]. Among the 159 patients, 122 (76.7%) did not achieve CR, and 37 (23.3%) achieved CR. The PRU value at 3 months was 167.80 ± 47.44 in patients with CR and 265.22 ± 21.56 in patients without CR. The ages of the CR-positive group were significantly higher than those of the CR-negative group (76.05 ± 7.54 vs. 71.02 ± 8.98 years, *p* = 0.002). Conversely, the CR-positive group had significantly lower alcohol consumption than the CR-negative group (10.8% vs. 32.8%, *p* = 0.009). Laboratory findings showed that the CR-positive group had a significantly lower glomerular filtration rate (GFR; 79.93 ± 15.05 vs. 86.45 ± 14.83, *p* = 0.021) and a significantly lower platelet count (212.08 ± 43.11 vs. 232.85 ± 56.33, *p* = 0.041) than the CR-negative group. No significant differences were observed in the other data (Table 1). A binominal logistic regression analysis was performed on age, alcohol consumption, platelet count, and ilaprazole use to identify the factors affecting CR, which showed a *p*-value of 0.1 or lower (Table 2). The results showed that age (adjusted odds ratio (aOR) 1.060, 95% confidence interval (CI) 1.011–1.112, *p* = 0.017) and alcohol consumption (aOR 0.331, 95% CI, 0.105–0.892; *p* = 0.048) were significantly different between the groups; however, the platelet count (aOR 0.993, 95% CI, 0.985–1.002; *p* = 0.125) and ilaprazole intake (aOR 0.527, 95% CI, 0.211–1.315; *p* = 0.170) were not significantly different.

### 3.2. Effects of Taking Ilaprazole on Clopidogrel Metabolism

Among the 210 patients, 66 (31.4%) took ilaprazole after hospitalization for stroke, while 144 (68.5%) did not. The proportion of alcohol drinkers was significantly higher in the ilaprazole group than in the nonilaprazole group (33.3% vs. 18.8%, *p* = 0.02). However, the proportion of patients with CR was not significantly different between the ilaprazole and nonilaprazole groups (15.1% vs. 27.4%). Additionally, the AST and ALT levels were not significantly different between the two groups. The PRU at three months after the onset of a stroke was not significantly different between the group taking ilaprazole and the group not taking ilaprazole (177.38 ± 58.46 vs. 197.02 ± 59.09) (Table 3).

## 4. Discussion

Anticoagulants or antiplatelet agents are important for secondary prophylaxis in ischemic stroke or cardiovascular disease. Moreover, they have been proven to be effective treatments. However, recurrent events may occur even after aspirin or clopidogrel treatment. These recurrent events can be caused by drug–drug interactions, which occur when different drugs affect one another, or caused by resistance to aspirin or clopidogrel [20]. Therefore, it is necessary to use drugs that do not influence clopidogrel metabolism. However, it is sometimes necessary to treat PPIs, which use the CYP450 pathway and are metabolized in the liver, similar to clopidogrel. In such cases, it is necessary to use PPIs that affect the metabolism of clopidogrel as little as possible. Among the PPIs, the use of esomeprazole and omeprazole with clopidogrel is not recommended because they use CYP2C19 and CYP3A4 as their main metabolites and may decrease the efficacy of clopidogrel [21,22]. However, ilaprazole does not influence clopidogrel metabolism because it does not use CYP2C19 and CYP3A4 as its main metabolic pathways [10,23]. We aimed to examine whether ilaprazole affected CR using VerifyNow, which easily computed the activity of clopidogrel and identified the factors affecting CR.

The study’s results showed that the mean age of the CR-positive group was higher than that of the CR-negative group. In other words, the results indicated that the effects of the drug decreased as the efficacy of clopidogrel decreased with increasing age. Approximately 15% of orally administered clopidogrel undergoes two steps of a metabolic pathway by the CYP enzyme family [8]. In the first step, the CYP2C19, CYP1A2, and CYP2B6 of CYP450 are involved in producing 2-oxoclopidogrel [8]. In the second step, CYP3A4, CYP2B6, CYP2C19, and CYP2C9 form a cis-thiol isomer [8]. Sotaniemi et al. reported that drug metabolism related to the CYP450 system in the liver decreased with age [24]. It is thought that the mean age of the CR patients was higher because the CYP450 function of older patients decreased to lower the ratio of transforming clopidogrel into its active form, which reduced the efficacy of clopidogrel.

The proportion of alcohol drinkers was higher in the CR-positive group than in the CR-negative group, which we think is associated with clopidogrel metabolism. Alcohol is a representative chemical that is metabolized in the liver and may cause drug–drug interactions with drugs metabolized in the liver [25]. When alcohol enters the body, it is metabolized in the GI tract [26]. It is then absorbed and metabolized to acetaldehyde by alcohol dehydrogenase and CYP2E1 [26]. Alcohol can also affect CYP2E1 and CYP3A4 [27]. Nicotinamide adenine dinucleotide (NAD+) is used when the CYP450 enzyme functions influence the redox reaction of reduced NAD+ to affect the reaction of drugs metabolized in the liver [27]. Moreover, carboxylesterase 1 (CES1) is present at high levels in most liver tissues [28], and 85% of the administered clopidogrel, not converted to the active form, is metabolized to clopidogrel carboxylate through hydrolysis by CES1 [8].

Alcohol is an inhibitor of CES1 and may affect other drugs by its interference with the action of CES1 in the liver [29]. In the presence of alcohol, clopidogrel is not hydrolyzed, but is converted to ethyl clopidogrel through transesterification [30,31]. However, as the amount of alcohol increases, CES1 is not metabolized, and the metabolism of both hydrolysis and transesterification decreases [30]. We think that the CR-negative group had a higher level of alcohol consumption because the patent form of clopidogrel increased due to alcohol consumption and the amount of clopidogrel that converted into the active form increased compared with the alcohol-free condition.

The GFR was also significantly different between the CR-negative and -positive groups. The GFR decreases with age and is a critical cause of chronic kidney disease (CKD) in the elderly [32]. Approximately 70% of the 80-year-old patients are affected by this condition [32]. The results of this study showed that the mean age of the CR-positive group was higher than that of the CR-negative group, which could explain why the GFR of the CR-positive group was lower than that of the CR-negative group. CKD is classified into five stages according to the GFR. Muller et al. confirmed the association between platelet inhibition and clopidogrel prescription among patients with CKD [33]. In that study, the functional damage caused by platelet inhibition was confirmed only in CKD stage 5. However, this study did not include any subjects classified as CKD stage 4 or CKD stage 5 based on the estimated GFR. Additionally, Maruyama et al. reported that among noncardiogenic ischemic stroke patients, those with CKD exhibited a higher CR [34]. However, the study was limited by its inability to compare CKD stages, and body weight was excluded from the GFR calculation. Elderly patients have a relatively higher possibility of dehydration than younger patients [35]. Moreover, the GFR of patients can be altered by other environmental factors, such as dehydration during the examination. Although the GFR was found to be a significant factor in this study, we did not test whether the GFR influenced CR positivity. Further studies are needed to confirm this hypothesis.

We divided patients taking clopidogrel after stroke into two groups: those taking and those not taking ilaprazole, and examined the effects of ilaprazole administration on platelet inhibition by clopidogrel. Most factors (e.g., sex and age) did not differ between the groups. However, the group taking ilaprazole had a significantly higher level of alcohol consumption. It is thought that in this retrospective study, alcohol-drinking patients had a higher possibility of gastritis in clinical practice, which made them express GI symptoms more frequently; consequently, significantly more alcohol-drinking patients were prescribed ilaprazole [36]. Additionally, if ilaprazole had an impact on clopidogrel metabolism, the group taking ilaprazole would have had a higher follow-up PRU than the group not taking ilaprazole after three months. However, the results of this study showed that the former had a lower follow-up PRU than the latter after three months, which confirmed that ilaprazole administration did not influence clopidogrel metabolism and platelet inhibition.

This study had several limitations. First, this was a retrospective study, and there may have been a selection bias in patient group selection. Second, we sampled patients at a single clinic, and the number of samples may have been too small to represent the entire patient group. Therefore, large-scale joint studies with other research institutes may be beneficial. Third, we did not examine the CYP2C19 polymorphism that may occur frequently in Asian populations during CR. This limitation may be overcome by a genetic study of CYP2C19. Lastly, we did not examine other drugs that may affect clopidogrel use in each patient. Patients could have taken drugs other than PPIs that might have affected clopidogrel metabolism using the CYP450 system. Examining this line of research may confirm the effects of clopidogrel on PPIs. Future studies should address these limitations.

## 5. Conclusions

This study reiterated that ilaprazole and clopidogrel can be simultaneously administered because they do not affect the metabolism of clopidogrel, as determined by using VerifyNow. We also confirmed that age and alcohol consumption affect clopidogrel metabolism. Further studies are needed to examine whether the GFR affects clopidogrel metabolism.

## Figures and Tables

**Table 1:** Comparison of baseline characteristics between patients with clopidogrel resistance versus patients without clopidogrel resistance.

Variable	Clopidogrel Resistance (−) (*n* = 122)	Clopidogrel Resistance (+) (*n* = 37)	*p*-Value
Male	73 (59.8)	17 (45.9)	0.135
Age	71.02 ± 8.98	76.05 ± 7.54	0.002
HTN	74 (60.7)	25 (67.6)	0.447
DM	33 (27.0)	13 (35.1)	0.342
HL	23 (18.9)	6 (16.2)	0.716
CAD	3 (2.5)	1 (2.7)	0.934
Alcohol drinking	40 (32.8)	4 (10.8)	0.009
Smoking	27 (22.1)	4 (10.8)	0.128
NIHSS	3 [2–5]	3 [2–4]	0.229
mRS	3 [2–4]	3 [2–3]	0.045
Recurrent stroke	8 (6.6)	3 (8.3)	0.715
Taking with ilaprazole	45 (36.9)	8 (21.6)	0.084
Laboratory findings			
Platelet	232.85 ± 56.33	212.08 ± 43.11	0.041
Sodium	139.37 ± 2.42	138.84 ± 3.29	0.368
Potassium	4.08 ± 0.40	4.04 ± 0.48	0.686
AST	30.73 ± 12.11	31.86 ± 12.37	0.62
ALT	26.47 ± 13.49	29.03 ± 15.24	0.328
BUN	16.63 ± 13.22	17.95 ± 6.36	0.56
Creatinine	0.78 ± 0.19	0.81 ± 0.26	0.483
GFR	86.45 ± 14.83	79.93 ± 15.05	0.021
Total cholesterol	173.70 ± 37.93	175.77 ± 43.09	0.785
Triglyceride	175.64 ± 190.43	186.14 ± 227.77	0.786
Low-density lipoprotein	114.23 ± 38.66	112.11 ± 45.90	0.786
High-density lipoprotein	45.48 ± 13.29	48.29 ± 12.89	0.273
PRU (3 months after)	167.80 ± 47.44	265.22 ± 21.56	<0.001
Inhibition (%)	31.05 ± 18.70	5.05 ± 6.69	<0.001

Data expressed as number (% column), mean ± standard deviation, or median [25–75 percentile range]. Nonparametric tests were performed for continuous variables that did not show normal distribution and are presented as median (25–75 percentile range). HTN, hypertension; DM, diabetes mellitus; HL, hyperlipidemia; CAD, coronary artery disease; NIHSS, National Institutes of Health Stroke Scale; mRS, modified Rankin scale; AST, aspartate aminotransferase; ALT, alanine aminotransferase; BUN, blood urea nitrogen; GFR, glomerular filtration rate; PRU, P2Y_12_ reaction unit.

**Table 2:** Factors associated with clopidogrel resistance.

Factor	Crude OR (95% CI)	*p*-Value	Adjusted OR (95% CI)	*p*-Value
Age	1.071 (1.023–1.121)	0.003	1.060 (1.011–1.112)	0.017
Alcohol	0.248 (0.082–0.750)	0.013	0.331 (0.105–0.892)	0.048
Platelet count	0.991 (0.983–0.999)	0.037	0.993 (0.985–1.002)	0.125
Ilaprazole	0.472 (0.199–1.121)	0.089	0.527 (0.211–1.315)	0.170

Results are expressed as odds ratios (ORs) and 95% confidence intervals (CIs). Variables with *p* < 0.1 by univariate analysis were entered into the multivariate analysis model. OR, odds ratio; CI, confidence interval.

**Table 3:** Comparison of baseline characteristics between patients taking versus not taking ilaprazole.

Factor	Without Ilaprazole (*n* = 144)	With Ilaprazole (*n* = 66)	*p*-Value
Male	77 (53.5)	39 (59.1)	0.447
Age	72.74 ± 9.07	72.55 ± 8.37	0.881
HTN	95 (66.0)	36 (54.5)	0.113
DM	43 (29.9)	16 (24.2)	0.4
HL	29 (20.1)	10 (15.2)	0.388
CAD	3 (2.1)	2 (3.0)	0.676
Alcohol drinking	27 (18.8)	22 (33.3)	0.02
Smoking	25 (17.4)	16 (24.2)	0.243
Clopidogrel resistance	29 (27.4)	8 (15.1)	0.084
NIHSS	3 [2–5]	3 [1–5]	0.259
mRS	3 [2–4]	3 [2–4]	0.628
Recurrent stroke	8 (6.2)	3 (5.4)	0.823
Laboratory findings			
Platelet	229.85 ± 57.37	224.39 ± 61.81	0.533
Sodium	137.63 ± 14.22	139.62 ± 2.42	0.259
Potassium	5.69 ± 14.19	4.11 ± 0.43	0.366
AST	31.05 ± 15.19	31.76 ± 12.08	0.739
ALT	26.65 ± 12.64	27.14 ± 15.65	0.809
BUN	17.60 ± 12.78	14.83 ± 4.65	0.09
Creatinine	0.79 ± 0.22	0.78 ± 0.20	0.569
GFR	8391 ± 14.57	85.70 ± 15.59	0.419
Total cholesterol	169.41 ± 36.58	176.67 ± 43.74	0.222
Triglyceride	188.22 ± 172.04	160.73 ± 228.74	0.348
Low-density lipoprotein	116.22 ± 87.35	118.03 ± 45.19	0.876
High-density lipoprotein	44.98 ± 12.58	47.65 ± 15.27	0.231
PRU (3 months after)	197.02 ± 59.09	177.38 ± 58.46	0.049
Inhibition (%)	23.71 ± 19.68	27.58 ± 20.54	0.25

Data are expressed as number (% column), mean ± standard deviation, or median [25–75 percentile range]. Nonparametric tests were performed for continuous variables that did not show normal distribution and are presented as median (25–75th percentile range). HTN, hypertension; DM, diabetes mellitus; HL, hyperlipidemia; CAD, coronary artery disease; NIHSS, National Institutes of Health Stroke Scale; mRS, modified Rankin scale; AST, aspartate aminotransferase; ALT, alanine aminotransferase; BUN, blood urea nitrogen; GFR, glomerular filtration rate; PRU, P2Y_12_ reaction unit.

## Data Availability

All data and material supporting our findings are contained within the manuscript.
